# Periapical bone response to bacterial lipopolysaccharide is shifted upon cyclooxygenase blockage

**DOI:** 10.1590/1678-7757-2018-0641

**Published:** 2019-06-03

**Authors:** Fernanda Regina Ribeiro-Santos, Geyson Galo da Silva, Igor Bassi Ferreira Petean, Maya Fernanda Manfrin Arnez, Léa Assed Bezerra da Silva, Lúcia Helena Faccioli, Francisco Wanderley Garcia Paula-Silva

**Affiliations:** 1 Universidade de São Paulo, Faculdade de Odontologia de Ribeirão Preto, Departamento de Clínica Infantil, Ribeirão Preto, São Paulo, Brasil.; 2 Universidade de Pernambuco, Arco Verde, Pernambuco, Brasil.; 3 Universidade de São Paulo, Faculdade de Ciências Farmacêuticas de Ribeirão Preto, Departamento de Análises Clínicas, Toxicológicas e Bromatológicas, Laboratório de Inflamação e Imunologia das Parasitoses, Ribeirão Preto, São Paulo, Brasil.

**Keywords:** Apical periodontitis, Lipopolysaccharide, Bone, Osteoclastogenesis, Indomethacin, Celecoxib, Cyclooxygenase

## Abstract

**Objectives:**

Infection, inflammation and bone resorption are closely related events in apical periodontitis development. Therefore, we sought to investigate the role of cyclooxygenase (COX) in osteoclastogenesis and bone metabolism signaling in periapical bone tissue after bacterial lipopolysaccharide (LPS) inoculation into root canals.

**Methodology:**

Seventy two C57BL/6 mice had the root canals of the first molars inoculated with a solution containing LPS from *E. coli* (1.0 mg/mL) and received selective (celecoxib) or non-selective (indomethacin) COX-2 inhibitor. After 7, 14, 21 and 28 days the animals were euthanized and the tissues removed for total RNA extraction. Evaluation of gene expression was performed by qRT-PCR. Statistical analysis was performed using analysis of variance (ANOVA) followed by post-tests (α=0.05).

**Results:**

LPS induced expression of mRNA for COX-2 (Ptgs2) and PGE_2_ receptors (*Ptger1, Ptger3* and *Ptger4*), indicating that cyclooxygenase is involved in periapical response to LPS. A signaling that favours bone resorption was observed because *Tnfsf11* (RANKL), *Vegfa, Ctsk*, *Mmp9, Cd36*, *Icam, Vcam1*, *Nfkb1* and *Sox9* were upregulated in response to LPS. Indomethacin and celecoxib differentially modulated expression of osteoclastogenic and other bone metabolism genes: celecoxib downregulated *Igf1r, Ctsk*, *Mmp9, Cd36*, *Icam1, Nfkb1*, *Smad3, Sox9*, *Csf3, Vcam1* and *Itga3* whereas indomethacin inhibited *Tgfbr1, Igf1r*, *Ctsk, Mmp9*, *Sox9, Cd36* and *Icam1*.

**Conclusions:**

We demonstrated that gene expression for COX-2 and PGE_2_ receptors was upregulated after LPS inoculation into the root canals. Additionally, early administration of indomethacin and celecoxib (NSAIDs) inhibited osteoclastogenic signaling. The relevance of the cyclooxygenase pathway in apical periodontitis was shown by a wide modulation in the expression of genes involved in both bone catabolism and anabolism.

## Introduction

Apical periodontitis is a localized immune response towards microorganisms present within the tooth root canal.^1^ Untreated inflammatory response in the periapical region leads to resorption of bone and dental tissue, culminating in the loss of the tooth.^2^ ^,^ ^3^ Gram-negative bacteria, a major component of infected root canals, present lipopolysaccharide (LPS) as a component of the outer cellular wall, which is a potent stimulator of immune response, released mainly during bacterial death.^4^ ^–^ ^6^ In the periapical area, LPS induces osteoclastogenic signaling, which culminates in bone resorption.^7^ ^,^ ^8^

Biochemical mediators are released locally to stimulate immune response during inflammatory events. Structural changes in arachidonic acid chains caused by cyclooxygenases (COX) lead to the synthesis of prostaglandins and thromboxanes.^9^ Cyclooxygenases are found in 2 isoforms, named COX-1 and COX-2. COX-1 is produced and constitutively expressed on most tissues, whereas COX-2 is produced in response to inflammatory stimuli and stimulates the production of prostaglandins involved in inflammatory reaction and is not usually found in most tissues under physiological conditions.^10^ ^,^ ^11^ Bacterial LPS induces prostaglandin E_2_ (PGE_2_) by macrophages,^12^ which activates protein-G coupled with EP1, EP2, EP3 and EP4 receptors in order to promote distinct cellular response.^13^ ^,^ ^14^

Cyclooxygenases are inhibited by nonsteroidal anti-inflammatory drugs (NSAIDs), a heterogeneous group of medications, which results in a reduction in the synthesis of prostaglandins, thus controlling pain, inflammation, and fever. NSAIDs might inhibit COX-1 or COX-2.^10^ selectively or specifically. The administration of a COX non-selective inhibitor (indomethacin) during the development of apical periodontitis resulted in lower intensity of the inflammatory infiltrate concomitant with less bone resorption,^15^ ^,^ ^16^ although the signaling involved in bone loss was not further investigated. Interestingly, *in vitro*, a COX-2 selective inhibitor (celecoxib) impaired osteoclast formation, suggesting prostaglandins have a role in bone resorption.^11^ ^,^ ^17^ We have previously demonstrated that the 5-lipoxygenase inhibitor is able to regulate RANK/RANKL/OPG in apical periodontitis;^18^ however, studies showing the effects of a COX-2 selective inhibitor and a non-selective COX-2 inhibitor on the regulation of RANK, RANKL, OPG and other genes involved in bone metabolism following root canal inoculation with LPS has not been reported. Because infection, inflammation and bone resorption are closely related events in apical periodontitis development, we hypothesized that blockage in the cyclooxygenase pathway would change the signaling involved in periapical bone response to bacterial lipopolysaccharide. For that reason, we used non-selective (indomethacin) and selective (celecoxib) cyclooxygenase inhibitors, mostly because indomethacin treatment impairs bone loss^15^ but presents several side effects,^10^ whereas celecoxib treatment is more specific but its effects on apical periodontitis development are not known.

Therefore, the aim of this study was to evaluate the expression of messenger mRNA of COX-2, PGE_2_ receptors, and osteoclastogenesis mediators (RANK, RANKL and OPG) in bone tissue after LPS inoculation into the root canals, and the effect of indomethacin and celecoxib in the blockage of the inflammatory response induced via COX expression. Moreover, we investigated the effects of COX inhibition in a wide range of genes involved in bone metabolism.

## Methodology

### Animals

Seventy two C57BL/6 6-week-old male mice (*Mus musculus*) were used for experimentation after IRB approval (#10.1.1618.53.0). Animals were anesthetized i.m. with 10% ketamine hydrochloride (150 mg/kg; National Pharmaceutical Chemistry Union Agener S/A, Embu-Guaçu, SP, Brazil) and xylazine (7.5 mg/kg; Dopaser, Labs Calier S/A, Barcelona, Spain) for operative procedures. Anesthesia was sustained throughout the experimental period.^18^

### Operative procedures

The animals were placed in a surgical table with a device for mandibular retraction. The right upper and lower first molars of each animal were used for LPS inoculation into the root canals.

Occlusal root canal accesses were gained with 1011 spherical diamond burs (KG Sorensen Ind. Com. Ltda., Barueri, SP, Brazil), root canals were located with a #06 K-file (Les Fils d’ Auguste Maillefer S/A, Ballaigues, Switzerland), and the radicular pulp tissue was removed.^18^ Then, 10 µL of LPS suspension (1.0 mg/mL) from *E. coli* 0127: B8 (L3129; Sigma-Aldrich Corp., St. Louis, MO, USA) were inoculated within root canals of each tooth by means of an automatic micropipette. The teeth were sealed with conventional glass ionomer cement (S.S. White Dental Articles Ltda, Rio de Janeiro, RJ, Brazil), mixed in accordance with the manufacturer's instructions. Tissues were collected 7, 14, 21 and 28 days after LPS inoculation into the root canals (n=6 teeth per group).

### Indomethacin and celecoxib treatment

Indomethacin (Cayman Chemical, Ann Arbor, MI, USA) solution was prepared in saline with 5% NaHCO_3_ and was given i.p. (5 mg/kg), one hour prior to LPS inoculation into the root canals, and daily throughout the experimental period. Celecoxib (Pfizer Inc., La Jolla, CA, USA) was prepared in ethanol and saline, being provided gavage (15 mg/kg) one hour prior to LPS inoculation into the root canals as well as daily throughout the experimental period. Healthy teeth from animals that did not receive medication were used as control.

### Total RNA extraction

Animals were euthanized by i.m. anaesthetic overdose, using 10% ketamine (300 mg/kg) and xylazine (30 mg/kg). Afterwards, the tissues containing bone and tooth were collected and frozen immediately. A pool of three teeth was used for RNA extraction as previously described.^18^ RNA was extracted from tissues using the RNeasy Mini kit (RNeasy^®^ Mini, Qiagen Inc., Valencia, CA, USA) and the samples were treated with DNAse I (RNase-Free DNase Set; Qiagen Inc.), according to manufacturer protocol. RNA integrity was analyzed using 1% agarose electrophoresis and quantity was estimated in NanoDrop 1000 (Thermo Fisher Scientific Inc., Wilmington, DE, USA) at 230, 260 and 280 nm wavelengths.

### Quantitative reverse transcriptase-polymerase chain reaction (qRT-PCR)

Complimentary DNA (cDNA) was synthesized from 1000 ng of total RNA using random primers (High Quality cDNA Reverse Transcriptase Kits, Applied Biosystems, Foster City, CA, USA). Aliquots of 2 µl of the total cDNA were amplified by qRT-PCR using the primers for Ptgs2 (COX-2; Mm00478374), *Ptger1* (EP1; Mm00443098), *Ptger2* (EP2; Mm00436051), *Ptger3* (EP3; Mm01316856), *Ptger4* (EP4; Mm00436053), *Tnfrsf11a* (RANK; Mm00437135), *Tnfsf11* (RANKL; Mm00441906) and *Tnfrsf11b* (OPG; Mm01205928) (TaqMan^®^ Gene Expression Assay, Applied Biosystems) in an Eppendorf Mastercycler^®^ ep Realplex (Eppendorf AG, Hamburg, Germany). *Gapdh* (GAPDH; Mm99999915) was used as reference gene. Amplification was done under the following conditions: denaturation at 95°C for 10 min; followed by 40 cycles of 95°C, 15 s and 60°C, 1 min. Reactions were performed in duplicate and relative quantification was performed using the ΔΔCt Method. Relative expression was compared using one-way ANOVA followed by Dunnett's test or two-way ANOVA followed by Bonferroni test (α=0.05).

### qRT-PCR array

Global evaluation of genes involved in bone metabolism was performed using a commercially available qRT-PCR *PCR Array* (Osteogenesis RT² Profiler PCR Array, PAMM-026; Qiagen Inc.). cDNA was synthesized from 200 ng of total RNA using the RT^2^ First Strand kit (Qiagen Inc.) to evaluate 84 genes involved in osteogenesis. qRT-PCR amplification was performed in an Eppendorf Mastercycler^®^ ep Realplex (Eppendorf AG) using SybrGreen. *Gusb, Hprt*, *Hsp90ab1, Actb* and *Gapdh* were used as reference genes. Amplification was done under the following conditions: denaturation at 95°C for 10 min; followed by 40 cycles of 95°C for 15 s and 60°C for 1 min. Dissociation curve was evaluated in order to determine the specificity of the primers, considering the melting temperature of the amplicon under the following conditions: temperature increase to 95°C for 15 s, followed by temperature decrease to 60°C for 15 s, gradually increasing the temperature to 95°C for 20 min and maintaining it at 95°C for 15 min. Relative quantification was performed using the ΔΔCt Method using healthy teeth as calibrators. Statistical analysis was performed using one-way ANOVA followed by Dunnett's test (α=0.05).

## Results

### LPS induces expression of mRNA for COX-2, PGE_2_ receptors and regulators of osteoclastogenesis

**Figure 1:**
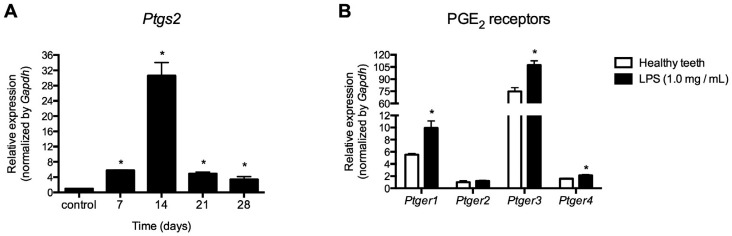
*Ptgs2* relative gene expression at 7, 14, 21 and 28 days following LPS inoculation into the root canals of mice (A) PGE_2_ receptors (*Ptger1, Ptger2*, *Ptger3, Ptger4*) were evaluated at 14 days following LPS inoculation into the root canals of mice (B) * p<0.05 compared to healthy teeth (control)

Inoculation of LPS into the root canals induced the expression of *Ptgs2*, mostly at 14 days following inoculation. After 21 days, *Ptgs2* expression decreased, although it remained higher than healthy teeth (Figure 1A). Because the peak of COX-2 expression was observed at 14 days, we investigated the expression of PGE_2_ receptors in this period. We observed that LPS induced expression of *Ptger1, Ptger3* and *Ptger4*, but exerted no influence on *Ptgs2,* which remained similar to healthy teeth (Figure 1B).

**Figure 2:**
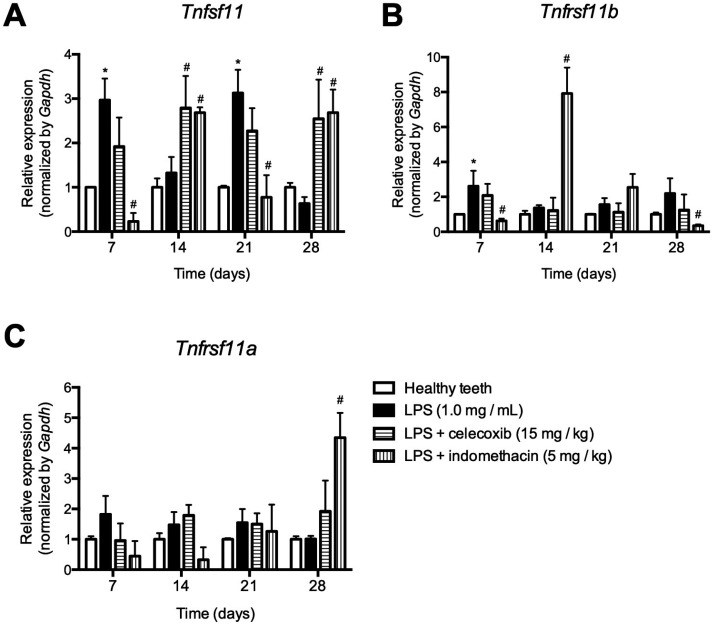
Relative expression of *Tnfsf11, Tnfrsf11b* and *Tnfrsf11a* which respectively encodes RANKL (A), OPG (B) and RANK (C), 7, 14, 21 and 28 days following LPS inoculation into the root canals of mice. After LPS inoculation, selective (celocoxib) or non-selective (indomethacin) COX-2 inhibitors were administered daily to the animals. * p<0.05 compared to healthy teeth; # p<0.05 compared to LPS inoculation alone

*Tnfsf11* expression, which encodes RANKL, increased at 7 and 21 days upon LPS stimulation but did not change at 14 and 28 days. *Tnfrsf11b* expression, which encodes OPG, increased at 7 days, but it was no different from healthy teeth after that. *Tnfrsf11a* expression, which encodes RANK, remained unchanged at all experimental periods (Figure 2).

### Indomethacin and celecoxib differentially modulate expression of osteoclastogenic genes

Administration of indomethacin, a COX-2 non-selective inhibitor, inhibited expression of RANK gene (Tnfrsf11a) at 7 and 14 days and exerted no effect at 21 days but stimulated expression at 28 days. RANKL gene (Tnfsf11) expression was inhibited at 7 and 21 days but increased at 14 and 28 days. The expression OPG gene (Tnfrsf11b) was stimulated at 14 and 21 days but did not change at 7 and 28 days (Figure 2).

On the other hand, administration of celecoxib, a COX-2 selective inhibitor, reduced the expression of RANK gene (Tnfrsf11a) at 7 days but increased at 14 and 28 days, with no change at 21 days compared to the LPS inoculation alone. RANKL gene (Tnfsf11) expression decreased at 7 and 21 days following treatment but increased at 14 and 28 days. The expression OPG gene (Tnfrsf11b) was not modulated by celecoxib treatment (Figure 2).

### *In vivo* LPS induces expression of genes involved in bone metabolism that are regulated by indomethacin and celecoxib

Because *Ptgs2* expression highly increased in early response to LPS inoculation into the root canal (14 days), a wide selection of genes involved in bone metabolism was investigated upon treatment with a selective (celecoxib) or non-selective COX-2 (indomethacin) inhibitor.

**Figure 3:**
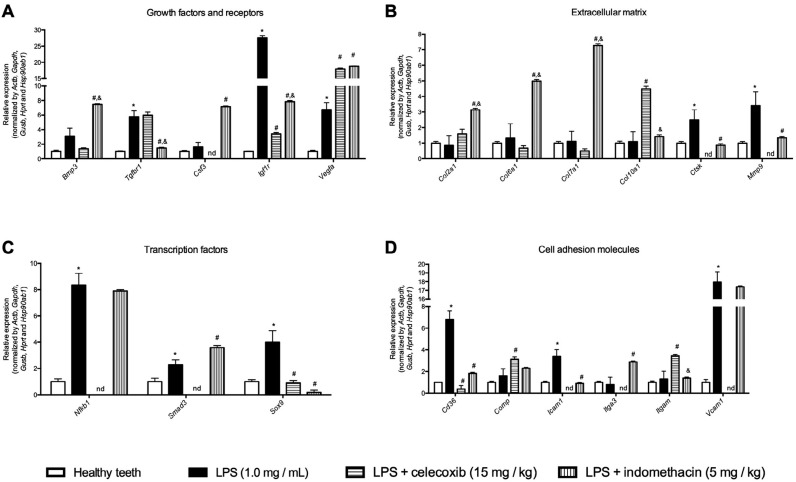
Relative expression of genes that encode growth factors (A), extracellular matrix (ECM) proteins (B), osteogenic transcription factors (C) and cellular adhesion molecules (D) 14 days following LPS inoculation into the root canals of mice, with or without selective (celocoxib) or non-selective (indomethacin) COX-2 inhibitors administered daily. * *p*<0.05 compared to healthy teeth; # *p*<0.05 compared to LPS inoculation alone; & *p*<0.05 compared to LPS + celocoxib (15 mg/kg)

At 14 days, LPS inoculation into the root canals increased mRNA expression of *Tgfbr1, Igf1r, Vegfa, Ctsk, Mmp9, Cd36, Icam, Vcam1, Nfkb1, Smad 3* and *Sox9*. Celecoxib administration downregulated *Igf1r, Ctsk, Mmp9, Cd36, Icam1, Nfkb1, Smad 3, Sox9, Csf3, Vcam1* and *Itga3* but upregulated *Tgfbr1, Vegfa, Col10a1, Comp* and *Itgam.* Indomethacin administration inhibited *Tgfbr1, Igf1r, Ctsk, Mmp9, Sox9, Cd36, Icam1,* and stimulated *Bmp3, Vegfa, Csf3, Col2a1, Col6a1, Col7a1, Itga3* and *Smad3* (Figure 3).

*Bmp5, Ambn*, *Ahsg, Enam* and *Tnf* were not detected in any group. *Alpl, Anxa5, Bgn, Bmp1, Bmp2, Bmp6, Bmpr1a, Cdh11, Col1a1, Col1a2, Col3a1, Col4a1, Col4a2, Col5a1, Col6a2*, *Col11a1, Col12a1*, *Col14a1, Dmp1*, *Egf, Fgf1*, *Fgf2, Fgfr1*, *Fgfr2, Fn1*, *Igf1, Itga1*, *Itga2b, Itga5*, *Itgb1, Mmp2*, *Mmp8, Mmp10*, *Msx1, Phex, Pdgfa*, *Runx2, Scarb1*, *Smad1, Smad2*, *Smad4, Serpinh1, Sost*, *Tfip11, Tgfb1, Tgfb3, Tgfbr2*, *Tuft1, Twist1, Vdr* and *Vegfb* were not modulated upon LPS stimulation or treatment with indomethacin or celecoxib (Table 1). *Bmp4, Bmpr1b, Csf2, Fgf3, Flt1, Gdf10, Tgfb2* and *Tgfbr3* were not detected in either positive or negative controls, i.e. LPS inoculation alone or health teeth, respectively. Therefore, further comparison between groups regarding the expression of the aforementioned genes could not be carried out.

**Table 1:** Gene expression not modulated during apical periodontitis development or upon treatment with indomethacin or celocoxib inhibitors for 14 days following root canal with LPS inoculation. Values depict mean (± standard deviation)

Symbol	Gene expression				p value
	Healthy teeth	LPS (1.0 mg/mL)	LPS + celecoxib (15 mg/kg)	LPS + indomethacin (5 mg/kg)	
*Alpl*	1.0 (± 0.12)	0.36 (± 1.98)	0.11 (± 0.13)	0.40 (± 0.12)	0.98
*Anxa5*	1.0 (± 0.10)	1.06 (± 1.62)	0.49 (± 0.15)	1.54 (± 0.09)	0.83
*Bgn*	1.0 (± 0.12)	1.32 (± 0.68)	nd	1.66 (± 0.11)	0.38
*Bmp1*	1.0 (± 0.10)	1.7 (± 0.91)	nd	1.83 (± 0.10)	0.37
*Bmp2*	1.0 (± 0.12)	0.27 (± 2.61)	nd	0.24 (± 0.19)	0.85
*Bmp6*	1.0 (± 0.15)	nd	nd	nd	–
*Bmpr1a*	1.0 (± 0.10)	0.79 (± 0.68)	0.96 (± 0.27)	0.49 (± 0.10)	0.56
*Cdh11*	1.0 (± 0.10)	1.19 (± 0.66)	0.18 (± 0.15)	0.41 (± 0.10)	0.11
*Col1a1*	1.0 (± 0.09)	1.59 (± 0.96)	0.28 (± 0.14)	0.92 (± 0.09)	0.20
*Col1a2*	1.0 (± 0.10)	1.65 (± 0.77)	0.30 (± 0.27)	0.54 (± 0.11)	0.10
*Col3a1*	1.0 (± 0.11)	0.76 (± 0.75)	0.03 (± 0.14)	0.27 (± 0.09)	0.66
*Col4a1*	1.0 (± 0.13)	1.44 (± 0.63)	nd	2.75 (± 0.10)	0.10
*Col4a2*	1.0 (± 0.11)	1.0 (± 0.68)	0.39 (± 0.36)	1.97 (± 0.10)	0.06
*Col5a1*	1.0 (± 0.11)	0.71 (± 0.63)	nd	0.66 (± 0.09)	0.65
*Col6a2*	1.0 (± 0.11)	1.02 (± 0.63)	0.32 (± 0.17)	0.37 (± 0.09)	0.18
*Col11a1*	1.0 (± 0.13)	0.92 (± 0.70)	0.08 (± 0.13)	0.19 (± 0.16)	0.13
*Col12a1*	1.0 (± 0.10)	0.68 (± 0.63)	0.18 (± 0.17)	0.13 (± 0.09)	0.15
*Col14a1*	1.0 (± 0.10)	0.26 (± 1.18)	0.13 (± 0.13)	0.04 (± 0.70)	0.45
*Dmp1*	1.0 (± 0.11)	1.31 (± 0.92)	nd	0.91 (± 0.11)	0.75
*Egf*	1.0 (± 0.11)	1.41 (± 1.79)	2.73 (± 0.28)	1.39 (± 0.09)	0.36
*Fgf1*	1.0 (± 0.10)	0.88 (± 0.63)	0.67 (± 0.18)	1.71 (± 0.10)	0.11
*Fgf2*	1.0 (± 0.10)	nd	0.74 (± 0.19)	0.60 (± 0.11)	0.13
*Fgfr1*	1.0 (± 0.07)	nd	nd	nd	–
*Fgfr2*	1.0 (± 0.10)	0.36 (± 0.73)	0.20 (± 0.72)	1.46 (± 0.11)	0.19
*Fn1*	1.0 (± 0.11)	1.34 (± 1.06)	1.15 (± 0.16)	0.97 (± 0.10)	0.89
*Igf1*	1.0 (± 0.09)	0.40 (± 0.64)	0.08 (± 0.14)	0.60 (± 0.09)	0.18
*Itga1*	1.0 (± 0.15)	nd	nd	0.67 (± 0.14)	0.15
*Itga2b*	1.0 (± 0.11)	0.50 (± 0.64)	0.80 (± 0.43)	0.73 (± 0.11)	0.67
*Itga5*	1.0 (± 0.12)	0.33 (± 0.71)	0.75 (± 0.19)	0.29 (± 0.11)	0.31
*Itgb1*	1.0 (± 0.11)	1.02 (± 1.32)	0.55 (± 0.58)	1.17 (± 0.10)	0.84
*Mmp2*	1.0 (± 0.10)	1.11 (± 0.94)	nd	0.92 (± 0.90)	0.96
*Mmp8*	1.0 (± 0.10)	1.83 (± 1.73)	nd	0.60 (± 0.10)	0.53
*Mmp10*	1.0 (± 0.11)	0.67 (± 0.77)	nd	0.76 (± 0.14)	0.77
*Msx1*	1.0 (± 0.10)	0.68 (± 0.84)	nd	1.83 (± 0.09)	0.19
*Phex*	1.0 (± 0.10)	0.97 (± 0.68)	0.69 (± 0.19)	0.04 (± 0.18)	0.16
*Pdgfa*	1.0 (± 0.12)	1.53 (± 1.22)	0.91 (± 0.13)	2.22 (± 0.10)	0.26
*Runx2*	1.0 (± 0.10)	1.56 (± 0.80)	nd	1.92 (± 0.06)	0.28
*Scarb1*	1.0 (± 0.10)	nd	nd	1.20 (± 0.09)	0.16
*Smad1*	1.0 (± 0.11)	0.92 (± 0.64)	1.39 (± 0.41)	0.39 (± 0.10)	0.22
*Smad2*	1.0 (± 0.11)	0.71 (± 1.75)	nd	1.16 (± 0.10)	0.90
*Smad4*	1.0 (± 0.12)	1.04 (± 0.99)	1.5 (± 0.17)	0.37 (± 0.10)	0.31
*Serpinh1*	1.0 (± 0.11)	0.87 (± 0.63)	0.69 (± 0.32)	0.35 (± 0.10)	0.41
*Sost*	1.0 (± 0.11)	0.60 (± 0.69)	0.89 (± 0.20)	0.83 (± 0.10)	0.75
*Tfip11*	1.0 (± 0.11)	1.4 (± 0.64)	1.69 (± 0.14)	1.49 (± 0.13)	0.34
*Tgfb1*	1.0 (± 0.12)	0.87 (± 0.63)	nd	1.16 (± 0.12)	0.75
*Tgfb3*	1.0 (± 0.11)	0.93 (± 0.63)	nd	0.37 (± 0.14)	0.32
*Tgfbr2*	1.0 (± 0.10)	0.5 (± 1.00)	3.58 (± 1.62)	0.42 (± 0.10)	0.07
*Tuft1*	1.0 (± 0.12)	0.84 (± 1.04)	1.08 (± 0.27)	2.08 (± 0.11)	0.23
*Twist1*	1.0 (± 0.11)	0.55 (± 1.01)	0.52 (± 0.14)	0.89 (± 0.10)	0.73
*Vdr*	1.0 (± 0.12)	0.82 (± 1.09)	0.29 (± 0.21)	0.97 (± 0.10)	0.60
*Vegfb*	1.0 (± 0.10)	nd	nd	0.68 (± 0.12)	0.10
nd - not detected					

## Discussion

We demonstrated that LPS inoculation into the root canals stimulated gene expression, encoding the COX-2 enzyme involved in the metabolism of arachidonic acid (*Ptgs2*) and PGE_2_ receptors, concomitantly to the expression modulation of osteoclastogenesis markers. The administration of a selective celecoxib) or non-selective (indomethacin) COX-2 inhibitor differentially regulated genes involved in bone metabolism.

The involvement of prostaglandins in apical periodontitis development has been previously demonstrated. Significantly higher concentrations of PGE_2_ were found in acute lesions when compared to chronic injuries,^16^ ^,^ ^19^ indicating a role for PGs in the pathogenesis of apical periodontitis. We observed a high *Ptgs2* gene expression at 14 days after LPS inoculation into root canals with subsequent decline, corroborating previous studies showing increased synthesis of prostaglandins in initial periods of response to contamination of root canals.^20^ ^,^ ^21^ Increased expression of *Ptger1, Ptger3* and *Ptger4* upon LPS inoculation into the root canals indicate that PGE_2_ might have been generated through arachidonic acid metabolism by COX-2 in order to signal through EP1, EP3 and EP4 receptors.^13^ ^,^ ^14^ ^,^ ^22^ Indeed, EP4 signaling under LPS stimulation induces osteoclast formation and periodontal bone destruction.^16^

COX2 derivatives are involved in periapical inflammatory response and daily administration of indomethacin results in a significantly reduced level of periapical bone resorption,^15^ although the role of COX metabolites in the regulation of bone loss in apical periodontitis is not well known. Here, we demonstrated that administration of indomethacin inhibited RANKL expression and increased OPG expression in early response to LPS, shedding light on a possible mechanism that resulted in the reduced bone resorption previously observed. In agreement, a COX-2 selective inhibitor (celecoxib) acts directly on circulating osteoclast precursors in order to inhibit osteoclast formation without exerting cytotoxic effect *in vitro.* ^11^ ^,^ ^23^ Previous studies have shown that early intervention in the COX-2 pathway modulates the gene expression of OPG, RANKL, RANK and other genes involved in the bone metabolism of induced arthritis in mice.^17^

In this study, we investigated the expression of genes regulating bone anabolism and catabolism in response to LPS. Several growth factors, cell differentiation, cellular adhesion, molecules and transcription factors were expressed 14 days in the apical periodontitis area upon LPS inoculation: *Tgfbr1, Igl1r, Vegfa, Ctsk, Mmp9, Nfkb1, Smad3, Sox9, Cd36, Icam1* and *Vcam1*. Vascular endothelial growth factors (*Vegf*) and their receptors control vasculogenesis and are also involved in bone resorption in apical periodontitis.^3^ ^,^ ^24^ In this study, we observed an increase in *Vegfa* upon LPS inoculation, with enhanced expression after administration of celecoxib and indomethacin. Apart from vasculogenesis, it has been speculated that immune cells communicate with each other and with endothelial cells in human apical periodontitis through VEGF since clustered ELA2, CD68, CD3 and CD19 immune cells are VEGFA, VEGFR-2, VEGFR-3 and VEGFD positive.^3^

Cell adhesion molecules such as *Vcam1* and *Icam1* play a role in leukocyte migration to the site of infection. *Icam1* deficiency results in compromised immune response reflected by enhanced periapical inflammation and bone resorption.^25^ In this study, we observed that indomethacin inhibited *Icam1* expression and celecoxib inhibited both *Icam1* and *Vcam1*.

Osteoclastogenesis markers *Mmp9* and *Ctsk* were stimulated in LPS inoculation but were inhibited by both celecoxib and indomethacin. MMP-9 is important for initiating the osteoclastic resorption process by removing the collagenous layer from the bone surface prior to mineral loss.^26^ In apical periodontitis, expression and activity of MMP-9 was found to be significantly higher than that in normal periodontal ligament tissue.^27^ ^,^ ^28^ Although it has been reported that MMP-9 knockout mice develop larger apical periodontitis than wild mice,^29^ those findings should be carefully interpreted since several MMPs might exert compensatory effect, colaborating with enhanced bone loss. Another protease, cathepsin K, is highly expressed by osteoclasts to promote degradation type I of collagen, resulting in bone destruction.^30^ Administration of cathepsin K inhibitor effectively downregulated apical periodontitis development and synthesis of proinflammatory cytokines.^31^

## Conclusions

We demonstrated that gene expression for COX-2 and PGE_2_ receptors is upregulated after LPS inoculation into the root canals and that early administration of indomethacin and celecoxib (NSAIDs) inhibits osteoclastogenic signaling. The relevance of the cyclooxygenase pathway in apical periodontitis is evidenced by a wide modulation in the expression of genes involved in both bone catabolism and anabolism. Our findings suggest that *in vivo* studies should be conducted to evaluate bone remodeling in apical periodontitis for a complimentary understanding of COX-related mechanisms in inflammatory periapical lesions. We envision that in future, local modulation of host response aimed at enhancing apical periodontitis resolution may supplement the traditional root canal treatment protocol.

## References

[1] Márton IJ, Kiss C (2000). Protective and destructive immune reactions in apical periodontitis. Oral Microbiol Immunol.

[2] Graves DT, Oates T, Garlet GP (2011). Review osteoimmunology and the host response in endodontic and periodontal lesions. J Oral Microbiol.

[3] Virtej A, Loes SS, Berggreen E, Bletsa A (2013). Localization and signaling patterns of vascular endothelial growth factors and receptors in human periapical lesions. J Endod.

[4] Graunaite I, Lodiene G, Maciulskiene V (2012). Pathogenesis of apical periodontitis: a literature review. J Oral Maxillofac Res.

[5] Nair PNR (2004). Pathogenesis of apical periodontitis and the causes of endodontic failures. Crit Rev Oral Biol Med.

[6] Ahmed GM, El-Baz AA, Hashem AA, Shalaan AK (2013). Expression levels of matrix metalloproteinase-9 and gram-negative bacteria in symptomatic and asymptomatic periapical lesions. J Endod.

[7] Yang J, Su N, Du X, Chen L (2014). Gene expression patterns in bone following lipopolysaccharide stimulation. Cell Mol Biol Lett.

[8] Chuang FH, Tsai CC, Chen JH, Chen KK, Chen YK, Lin YC (2012). Long-term sequential receptor activator of NF-κB ligand (RANKL) and osteoprotegrin (OPG) expression in lipopolysaccharide-induced rat periapical lesions. J Oral Pathol Med.

[9] Kovác J, Kovác D (2011). Histopathology and etiopathogenesis of chronic apical periodontitis-periapical granuloma. Epidemiol Mikrobiol Imunol.

[10] Fitzpatrick FA (2004). Cyclooxygenase enzymes: regulation and function. Curr Pharm Des.

[11] Kawashima M, Fujikawa Y, Itonaga I, Takita C, Tsumura H (2009). The effect of selective cyclooxygenase-2 inhibitor on human osteoclast precursors to influence osteoclastogenesis *in vitro*. Mod Rheumatol.

[12] Xiao L, Ornatowska M, Zhao G, Cao H, Yu H, Deng J (2012). Lipopolysaccharide induced expression of microsomal prostaglandin E synthase-1 mediates late-phase PGE_2_ production in bone marrow derived macrophages. Plos One.

[13] Narumiya S, Sugimoto Y, Ushikubi F (1999). Prostanoid receptors: structures, properties, and functions. Physiol Rev.

[14] Sugimoto Y, Narumiya S (2007). Prostaglandin E receptors. J Biol Chem.

[15] Oguntebi BR, Barker BF, Anderson DM, Sakumura J (1989). The effect of indomethacin on experimental dental periapical lesions in rats. J Endod.

[16] Oka H, Miyauchi M, Furusho H, Nishihara T, Takata T (2012). Oral administration of prostaglandin E(2)-specific receptor 4 antagonist inhibits lipopolysaccharide-induced osteoclastogenesis in rat periodontal tissue. J Periodontol.

[17] Naidu VG, Babu KR, Thwin MM, Satish RL, Kumar PV, Gopalakrishnakone P (2013). RANKL targeted peptides inhibit osteoclastogenesis and attenuate adjuvant induced arthritis by inhibiting NF-kB activation and down regulating inflammatory cytokines. Chem Biol Interact.

[18] Paula-Silva FW, Petean IB, Silva LA, Faccioli LH (2016). Dual role of 5-lipoxygenase in bacterial-induced apical periodontitis. J Endod.

[19] Alptekin NO, Ari H, Haliloglu S, Alptekin T, Serpek B, Ataoglu T (2005). The effect of endodontic therapy on periapical exudate neutrophil elastase and prostaglandin-E2 levels. J Endod.

[20] Okiji T, Morita I, Sunada I, Murota S (1989). Involvement of arachidonic acid metabolites in increases in vascular permeability in experimental dental pulpal inflammation in the rat. Arch Oral Biol.

[21] Ohkura N, Shigetani Y, Yoshiba N, Yoshiba K, Okiji T (2014). Prostaglandin transporting protein-mediated prostaglandin E2 transport in lipopolysaccharide-inflamed rat dental pulp. J Endod.

[22] Kawahara K, Hohjoh H, Inazumi T, Tsuchiya S, Sugimoto Y (2015). Prostaglandin E2-induced inflammation: Relevance of prostaglandin E receptors. Biochim Biophys Acta.

[23] Igarashi K, Woo JT, Stern PH (2002). Effects of a selective cyclooxygenase-2 inhibitor, celecoxib, on bone resorption and osteoclastogenesis *in vitro*. Biochem Pharmacol.

[24] Bletsa A, Virtej A, Berggreen E (2012). Vascular endothelial growth factors and receptors are up-regulated during development of apical periodontitis. J Endod.

[25] De Rossi A, Rocha LB, Rossi MA (2008). Interferon-gamma, interleukin-10, intercellular adhesion molecule-1, and chemokine receptor 5, but not interleukin-4, attenuate the development of periapical lesions. J Endod.

[26] Delaissé JM, Engsig MT, Everts V, del Carmen Ovejero M, Ferreras M, Lund L (2000). Proteinases in bone resorption: obvious and less obvious roles. Clin Chim Acta.

[27] Paula-Silva FW, D'Silva NJ, Silva LA, Kapila YL (2009). High matrix metalloproteinase activity is a hallmark of periapical granulomas. J Endod.

[28] Paula-Silva FW, Silva LA, Kapila YL (2010). Matrix metalloproteinase expression in teeth with apical periodontitis is differentially modulated by the modality of root canal treatment. J Endod.

[29] Wan C, Yuan G, Yang J, Sun Q, Zhang L, Zhang J (2014). MMP-9 deficiency increased the size of experimentally induced apical periodontitis. J Endod.

[30] Saftig P, Hunziker E, Wehmeyer O, Jones S, Boyde A, Rommerskirch W (1998). Impaired osteoclastic bone resorption leads to osteopetrosis in cathepsin-K-deficient mice. Proc Natl Acad Sci U S A.

[31] Suzuki N, Takimoto K, Kawashima N (2015). Cathepsin K inhibitor regulates inflammation and bone destruction in experimentally induced rat periapical lesions. J Endod.

